# Anthracycline-Free Neoadjuvant Treatment in Patients with HER2-Positive Breast Cancer: Real-Life Use of Pertuzumab, Trastuzumab and Taxanes Association with an Exploratory Analysis of PIK3CA Mutational Status

**DOI:** 10.3390/cancers14123003

**Published:** 2022-06-18

**Authors:** Azzurra Irelli, Alessandro Parisi, Carla D’Orazio, Tina Sidoni, Silvia Rotondaro, Leonardo Patruno, Francesco Pavese, Alberto Bafile, Valter Resta, Laura Pizzorno, Virginia Ciuffetelli, Antonella Dal Mas, Giuseppe Calvisi, Alessandra Di Sibio, Anna Marzullo, Veronica Zelli, Chiara Compagnoni, Alessandra Tessitore, Edoardo Alesse, Corrado Ficorella, Alessio Cortellini, Katia Cannita

**Affiliations:** 1 Medical Oncology Unit, Department of Oncology, AUSL 04 Teramo, 64100 Teramo, Italy; kcannita@gmail.com; 2 Department of Life, Health and Environmental Sciences, University of L’Aquila, 67100 L’Aquila, Italy; alexparis@hotmail.it; 3 Department of Oncology, Università Politecnica delle Marche, Azienda Ospedaliero-Universitaria Ospedali Riuniti di Ancona, 60126 Ancona, Italy; 4 Medical Oncology, St. Salvatore Hospital, 67100 L’Aquila, Italy; doraziocarla@gmail.com (C.D.); tina_sidoni@yahoo.it (T.S.); silviaroto@libero.it (S.R.); patruno.leonardo@gmail.com (L.P.); francesco.pavese@outlook.it (F.P.); corrado.ficorella@univaq.it (C.F.); 5 Breast Unit, St. Salvatore Hospital, 67100 L’Aquila, Italy; albertobafile@alice.it (A.B.); vresta@asl1abruzzo.it (V.R.); laura.pizzorno@gmail.it (L.P.); 6 Pathology Unit, St. Salvatore Hospital, 67100 L’Aquila, Italy; virant@alice.it (V.C.); antonella.dalmas@gmail.com (A.D.M.); gcalvisi@asl1abruzzo.it (G.C.); 7 Radiology Unit, St. Salvatore Hospital, 67100 L’Aquila, Italy; adisibi@hotmail.com; 8 Nuclear Medicine Unit, St. Salvatore Hospital, 67100 L’Aquila, Italy; anna.marzullo@virgilio.it; 9 Department of Biotechnological and Applied Clinical Sciences, University of L’Aquila, Via Vetoio, 67100 L’Aquila, Italy; veronica.zelli@univaq.it (V.Z.); chiara.compagnoni@univaq.it (C.C.); alessandra.tessitore@univaq.it (A.T.); edoardo.alesse@univaq.it (E.A.); 10 Department of Surgery & Cancer, Imperial College London, Hammersmith Hospital, Du Cane Road, London W12 0HS, UK; alessiocortellini@gmail.com

**Keywords:** neoadjuvant treatment, HER2-positive, breast cancer, pertuzumab, trastuzumab, anthracyclines, taxanes, PIK3CA

## Abstract

**Simple Summary:**

This retrospective observational study aims at highlighting the response to pertuzumab, trastuzumab and docetaxel treatment (THP), without using anthracycline, in patients with HER2-positive early breast cancer. We add evidence to the suitability of THP in real life to reach higher pathological complete response rates, with a good safety profile for patients. An exploratory analysis of the mutational status of PIK3CA was performed as well, and 21% mutated samples were identified, with overall higher pCR rates in PIK3CA mutant patients and, particularly, in those who were THP-treated. Our original results open the door to further studies focused on more in-depth analysis of the molecular and clinical features related to the response to anthracycline-free neoadjuvant treatment in HER2-positive early breast cancer patients.

**Abstract:**

HER2 is considered one of the most traditional prognostic and predictive biomarkers in breast cancer. Literature data confirmed that the addition of pertuzumab to a standard neoadjuvant chemotherapy backbone (either with or without anthracyclines), in patients with human epidermal growth factor receptor 2 (HER2)-positive early breast cancer (EBC), leads to a higher pathological complete response (pCR) rate, which is known to correlate with a better prognosis. In this retrospective analysis, 47 consecutive patients with HER2-positive EBC received sequential anthracyclines and taxanes plus trastuzumab (ATH) or pertuzumab, trastuzumab and docetaxel (THP). Despite the limited sample size, this monocentric experience highlights the efficacy (in terms of pCR) and safety of THP in the neoadjuvant setting of HER2-positive EBC as an anthracycline-free approach. Given the role of PIK3CA as a prognostic and therapeutic target in breast cancer, tumors were also analyzed to assess the PIK3CA mutational status. Thirty-eight out of forty-seven patients were evaluated, and PIK3CA variants were identified in 21% of tumor samples: overall, one mutation was detected in exon 4 (2.6%), two in exon 9 (5.3%) and four in exon 20 (10.5%). Of note, one sample showed concurrent mutations in exons 9 (codon 545) and 20 (codon 1047). Among patients reaching pCR (n = 13), 38.5% were PIK3CA mutants; on the other hand, among those lacking pCR (n = 25), just 12% showed PIK3CA variants. Regarding THP-treated mutant patients (n = 5), 80% reached pCR (three hormone-receptor-negative, one hormone-receptor-positive). Interestingly, the only patient not achieving pCR had a tumor with two co-occurring PIK3CA mutations. In conclusion, this study provides new evidence about the efficacy and good safety profile of THP, compared to the ATH regimen, as an anthracycline-free neoadjuvant treatment of HER2-positive EBC. Further studies on larger/multicentric cohorts are planned for more in-depth analysis to confirm our molecular and clinical results.

## 1. Introduction

Human epidermal growth factor receptor 2 (HER2) overexpression is present in approximately 20% of invasive breast cancers. It is considered as an adverse prognostic factor and a predictor of anti-HER2 therapies response [1].

It is known that trastuzumab, combined with a neoadjuvant chemotherapy regimen including anthracycline and a taxane, achieves a pathological complete response (pCR) of 38%, compared to 19% obtained with chemotherapy only (*p* = 0.001) in patients with HER2-positive breast cancer (BC) [2].

The double blocking of HER2 (pertuzumab and trastuzumab) further increases the pCR rate to over 45% [3,4,5,6]. A recent meta-analysis demonstrated that the use of anthracyclines does not lead to better results, while it increases the incidence of side effects compared to anthracycline-free regimens [7,8]. This evidence reflects the TRAIN2 trial, which directly compared an anthracycline-containing regimen with a non-anthracycline-containing regimen [9]. The TRAIN2 study showed better outcomes in the anthracycline-free treatment arm in the high-risk group with four or more lymph nodes involved.

No prospective randomized study has demonstrated an outcome benefit from adding anthracyclines to taxane plus trastuzumab chemotherapy for HER2-positive breast cancer in the neoadjuvant and adjuvant settings. In fact, in 2021, the guidelines of the National Comprehensive Cancer Network removed anthracycline-based therapy from the list of recommended regimens for the treatment of early-stage HER2-positive breast cancer [10].

The pCR was considered by the FDA as a surrogate for long-term outcome (i.e., disease-free survival and overall survival) in randomized studies on neoadjuvant therapy for the treatment of early-stage breast cancer. On the other hand, the pCR should not be used as a surrogate endpoint for the accelerated approval of drugs tested in the neoadjuvant treatment of breast cancer. However, the strong association between pCR and overall survival is the best available indicator to predict the residual risk of relapse after neoadjuvant therapy in patients [11].

Starting from these considerations, we designed an observational retrospective study aimed at comparing in real life, in terms of pCR, sequential anthracyclines and taxanes plus trastuzumab (ATH) with pertuzumab, trastuzumab plus taxanes (THP) as neoadjuvant treatment in patients with HER2-positive early BC (EBC). PIK3CA is the most frequently mutated oncogene in all cancers [12]. Its codes for the catalytic subunit p110α, which associates to the regulatory p85α subunit to form the phosphoinositide 3-kinase alpha (PI3Kα) complex. PI3Kα operates in the PI3K/Akt/mTOR signaling pathway, playing a role in cell growth and proliferation after activation by membrane receptor tyrosine kinase (RTKs) binding. It is known that PIK3CA gene mutations, mainly at the level of hotspots in exons 9 (codons 542 and 545) and 20 (codon 1047), confer constitutive oncogenic pathway activation in different types of cancer, including breast cancer [13], for which it is considered a therapeutic target as well [14]. Furthermore, the PI3K pathway appears to be associated with resistance to anti-HER2 therapies [15,16], and PIK3CA mutations have been described in a variable percentage of HER2-positive breast cancer [17]. Several studies on neoadjuvant treatment of patients with HER2-positive breast cancer have shown that PIK3CA mutations can be associated with lower pCR rates [18], more marked in estrogen receptor (ER)-positive cancer [19], and shorter disease-free survival (DFS) [17]. However, the association between PIK3CA mutations and HER2-positive BC is still unclear, since previous analyses have either correlated PIK3CA mutations with favorable clinical outcomes or found no significant impact [16,17,20,21,22,23,24,25,26]. Thus, in this study, an exploratory analysis of PIK3CA mutational status was performed as well in the same patients treated with the ATH or THP scheme.

Our study adds new evidence to the real use, in terms of efficacy and safety, of THP as an anthracycline-free therapeutic approach suitable for HER2-positive EBC, by considering the potential role, still controversial, of PIK3CA mutational status as well.

## 2. Materials and Methods

### 2.1. Study Design and Patient Recruitment

This retrospective analysis (121 months follow-up) was focused on evaluating the activity and safety of sequential anthracyclines/taxanes plus trastuzumab in comparison to pertuzumab/trastuzumab plus taxanes as neoadjuvant medical treatments in patients with HER2-positive stage II–III BC.

Between November 2010 and December 2020, 91 consecutive patients with EBC completed the pre-planned neoadjuvant medical treatment, followed by breast surgery at the Medical Oncology Department of the St. Salvatore Hospital, University of L’Aquila, Italy.

Patients with luminal A-like BC, luminal B-like HER2-negative BC, triple-negative BC and with HER2-positive BC not treated with THP nor sequential ATH were excluded from the present study.

Eligibility criteria were: age ≥ 18 years; histologically confirmed diagnosis of HER2-positive (i.e., 3+ result by immunohistochemistry (IHC), or 2+ result by IHC and positive result by fluorescence in situ hybridization (FISH) assessed as per local routine clinical practice) early-stage BC; received neoadjuvant combination treatment with either 4 cycles of epirubicin (90 mg/mq) plus cyclophosphamide (600 mg/mq) d1 q14 followed by 6 cycles of docetaxel (60 mg/mq) plus trastuzumab (at a loading dose of 6mg/kg, and 4 mg/kg thereafter) d1 q14 (from November 2010 to March 2017), or with 6 cycles of pertuzumab (at a loading dose of 840 mg, and 420 mg thereafter) plus trastuzumab (at a loading dose of 8 mg/kg, and 6 mg/kg thereafter) plus docetaxel (75 mg/mq) d1 q21 (from April 2017 to December 2020) (Table 1).

The above neoadjuvant treatment schemes have been prescribed according to national and international guidelines [27,28].

Patients’ disease was evaluated by instrumental examinations: ultrasound at baseline and every three cycles; mammography and/or magnetic resonance imaging were performed at baseline and at the end of the neoadjuvant treatment.

Surgical resection was scheduled within 3 to 6 weeks from the last cycle of neoadjuvant treatment.

The primary endpoint was the rate of total pathological complete response (pCR), defined as the absence of invasive residual tumor in the breast and in axillary lymph nodes (ypT0/is ypN0), assessed by two defined pathologists at our Centre.

The secondary study endpoint was the incidence of treatment-related adverse events (AEs).

AEs experienced during treatment were reported for the two regimens, registered according to National Cancer Institute Common Terminology Criteria (NCI-CTC) for AEs (version 4 up to January 2018, version 5 from January 2018), and grouped according to severity (G1–2 and G3–4). Patients were evaluated to assess safety at every cycle by considering two cardiac biomarker assays (Troponin I and N-terminal pro-brain natriuretic peptide). Patients underwent echocardiography and electrocardiography every three cycles.

An ancillary and exploratory analysis was conducted to compare activity results with the PIK3CA status, assessed on the diagnostic tissue biopsy, available for 38 tumor samples.

All patients alive at the time of data collection provided informed consent to participate to this retrospective observational non-interventional study. The procedures followed were in accordance with the precepts of Good Clinical Practice and the Declaration of Helsinki. The study was approved by the Local Ethical Committee of our Centre (Prot. No. 32865/2018).

Data cut-off period was June 2021.

### 2.2. PIK3CA Genotyping

PIK3CA genotype was determined by allele-specific Real-Time PCR. DNA was extracted from FFPE tissues by using the Zymo Quick DNA FFPE Kit, according to the manufacturer’s instructions. Afterwards, the CE-IVD EasyPGX PIK3CA kit (Diatech Pharmacogenetics; 60035 Jesi, Italy) was used for mutation analysis, according to the manufacturer’s instructions. The highly sensitive assays (0.5% < LOD < 2%) allow the detection of approximately 90% of all the PIK3CA variants described in breast cancer, based on the Cosmic Database (https://cancer.sanger.ac.uk/cosmic, accessed on 12 December 2021), in exons 4, 7, 9 and 20.

### 2.3. Statistical Analysis

Baseline patient characteristics were reported with descriptive statistics (means, medians and proportions) as appropriate. The χ2 and Fisher’s exact tests were used to compare categorical variables between the two cohorts.

## 3. Results

### 3.1. Patients’ Characteristics

In total, 47 consecutive patients with HER2-positive early-stage BC were treated, 24 (51.1%) with sequential anthracyclines and taxanes plus trastuzumab (ATH), 23 (48.9%) with pertuzumab, trastuzumab and taxanes (THP). Patients’ features are summarized in Table 2.

The median patient age was 49 years (34–79): <40 years, 5 (10.6%); ≥40 years, 42 (89.4%). Twenty-eight (59.6%) patients were premenopausal. All patients had Eastern Cooperative Oncology Group Performance Status (ECOG PS) 0–1. Only one (2.1%) patient presented with secondary Cumulative Illness Rating Scale (CIRS). Ten patients (21.3%) had cardiovascular comorbidities. Forty-two patients (89.4%) had invasive ductal cancer and three patients (6.4%) had invasive lobular cancer. In addition, hormone positivity was found in 26 (55.3%) patients, and hormone negativity was found in 21 (44.7%). A total of 26 patients (55.3%) had grade 3 disease, and a total of 36 patients (76.6%) had Ki-67 ≥ 20%. One (2.1%), twenty-six (55.3%), seven (14.9%) and thirteen (27.7%) patients had T1, T2, T3 and T4 tumors, respectively. Nine (19.1%), twenty-eight (59.6%), nine (19.1%) and one (2.1%) tumors were classified as N0, N1, N2 and N3, respectively. The modalities of breast surgery were: mastectomy, 25 (53.2%); quadrantectomy, 22 (46.8%); sentinel node biopsy, 35 (74.5%); axillary dissection, 12 (25.5%). Intravenous/subcutaneous adjuvant therapy was administered to 16 (34%) patients with pertuzumab plus trastuzumab, 23 (48.9%) patients with trastuzumab and 5 (10.6%) patients with TDM-1. All patients with an ER/PgR positive tumor received adjuvant hormone therapy: 26 (55.3%).

After the completion of the neoadjuvant treatment, all patients underwent surgery three to five weeks after the last cycle of systemic therapy, after a median of 35 days.

Median follow-up was 18 months.

### 3.2. Pathological Response Analysis and PIK3CA Genotyping

The rate of pCR was 68.4% and 31.6% in BC patients treated with THP and ATH, respectively (*p* = 0.028). The main predictor of major response was hormone-receptor status (*p* = 0.036) (Table 3).

Among the 47 enrolled patients with HER2-positive EBC treated with THP or ATH, 38 patients were also evaluated for PIK3CA mutational status. Tissue samples from 8 out of 38 patients evaluated (21%) showed PIK3CA variants. Overall, there were one variant (2.6%) in exon 4, two variants (5.3%) in exon 9, four variants (10.5%) in exon 20, and one patient carried two co-occurring variants in exon 9 and 20 (Table 4).

Regarding the association between pathological response achievement and PIK3CA mutant (mut) or wild-type (wt) status, the statistical analysis did not reveal a significant difference. However, the presence of PIK3CA variants seems to be associated with higher pCR rates, being observed in 38.5% (5 out of 13) of patients with pCR and in 12% (3 out of 25) of those lacking pCR.

Five patients with PIK3CA mutant tumors were treated with the THP regimen, and four of them (80%) achieved pCR: three with a hormone-receptor-negative tumor, and one with a hormone-receptor-positive tumor. The patient not achieving pCR had a tumor carrying a double PIK3CA mutation.

Three out of eight patients with a PIK3CA mutated tumor, were treated with the ATH regimen: one patient obtained pCR; two patients did not.

### 3.3. Safety Analysis

The major observed adverse events (grade, G ≥ 3) were: G4 neutropenia (8.3% in the ATH group vs. 0 in the THP group), G3 neutropenia (16.7% in the ATH group vs. 0 in the THP group), G3 diarrhea (4.2% in the ATH group vs. 8.7% in the THP group), G3 asthenia (8.3% in the ATH group vs. 0 in the THP group), G3 hypertransaminasemia (8.3% in the ATH group vs. 0 in the THP group).

No grade ≥2 cardiac toxicity was observed. (Table 5 and Table 6)

## 4. Discussion

To date, there are limited available data on the efficacy of the neoadjuvant use of pertuzumab in addition to trastuzumab and chemotherapy in clinical practice [6].

In this study, a higher pCR rate was achieved by using the combination of the HER2 double-block and taxane (THP) versus ATH.

In particular, the pCR achieved by the THP group was 68.4% higher than that detected in the THP arm of the NeoSphere (45.8%), [29] Neopetra (59.3%), [30] Berenice (61.8%), [5] and GeparSepto (66.2%) studies, [4] but was in accordance with the pCR rate shown by the TRAIN-2 study (68%) [9].

In particular, the Neopetra study offers an example of retrospective experience focused on the real-word efficacy evaluation, in terms of pCR, of pertuzumab and trastuzumab plus chemotherapy, even with single-agent taxane, with a good safety profile [30].

In our study, the major adverse events observed (G ≥ 3) were neutropenia, leukopenia, asthenia and hypertransaminasemia in the ATH group, and diarrhea in the THP group. No patient treated with THP presented grade 3 or higher hematological toxicity, in particular, no leuko-neutropenia due to the use of granulocyte growth factors.

Literature data display that the most frequent grade 3 or higher toxicities were neutropenia, febrile neutropenia and leukopenia, particularly in patients treated with ATH [29].

Furthermore, the only grade 3 or higher toxicity in the THP group was diarrhea (8.7%), also described, although with lower frequency (4.2%), in the ATH group.

Based on studies on the use of pertuzumab in the metastatic (CLEOPATRA study) and neoadjuvant (NeoSphere and TRYPHAENA studies) settings, it appears that diarrhea of all degrees is a frequent adverse event associated with pertuzumab. Furthermore, the risk of all grades and severe diarrhea, associated with the use of neoadjuvant pertuzumab for HER2-positive breast cancer, was increased in clinical practice with respect to that observed in trials [31,32].

Additionally, it is described that cardiotoxicity is associated with both anthracyclines and PH, and the combination of these three drugs can cause an increased risk of cardiotoxicity.

In the meta-analysis by Mantarro et al., severe cardiotoxicity occurred in 2.62% of early BC [33].

In the CLEOPATRA study (metastatic setting), the incidence of cardiac adverse events (all grades) was 16.4% in the placebo arm and 14.5% in the pertuzumab arm, with left ventricular systolic dysfunction (LVSD) reported as the most frequent event, with detected left ventricular ejection fraction (LVEF) decreased more than 10%, with respect to baseline, up to less than 50%, in accordance to that previously reported in the neoadjuvant setting [9]. The combination of pertuzumab plus trastuzumab and docetaxel did not increase the incidence of cardiac adverse events, including LVSD, compared to the control arm in the HER2-positive metastatic BC. Most cardiac adverse events were reversible [34].

No grade ≥2 cardiac toxicity was observed in our study. Grade 1 cardiac toxicity was observed in the ATH group.

Therefore, the long-term and potentially life-threatening side effects of anthracyclines further draw attention to their use, particularly in HER2-positive breast cancer, for which new anti-HER2 drugs can be available [10].

Indeed, in addition to long-term cardiotoxicity, patients treated with epirubicin and cyclophosphamide for non-metastatic breast cancer have increased risks of myelodysplastic syndrome and acute myeloid leukemia [35,36].

Given the role of PIK3CA in breast cancer pathogenesis and as a predictive biomarker as well, we performed a mutational analysis focused on the most (approximately 90%) common PIK3CA variants described in breast cancer. We obtained 21% overall mutation frequency, in line with the literature, where it is reported that, for HER2-positive patients, depending on the molecular subtype, PIK3CA mutation frequency ranges from 12% to 39% [19,37,38,39,40,41]. Moreover, we detected higher variant frequency at the level of PIK3CA codon H1047, in accordance with data from BC patients [42]. Interestingly, one sample showed two co-occurring mutations in exons 9 (codon 545) and 20 (codon 1047), coding for the helical and kinase protein domain, respectively. Multiple PIK3CA mutations were described in BC by Vasan et al. [43], in a percentage ranging from 12 to 15% of PIK3CA mutant genomes. In this regard, the presence of double variants, especially in cis, seems to be associated with improved oncogenicity and sensitivity to PI3K inhibitors. To date, results regarding the association between PIK3CA mutations and clinical outcomes in a neoadjuvant setting are not entirely consistent, suggesting unfavorable clinical outcomes in terms of pCR rate and shorter DFS [17,18,19,41,44,45,46,47,48], favorable clinical outcomes [20,22] or no clinical impact [21,22,23,24,25,26,49].

In this study, we did not detect a statistically significant association between pCR rate and PIK3CA mutational status, even though our data may suggest that, overall, the presence of PIK3CA mutations could be associated with a higher pCR rate, mainly shown by patients who were THP-treated. Furthermore, among those receiving THP, the patient carrying the double PIK3CA mutation was the only one not reaching pCR. However, due to the small size of the samples, further analyses on larger/multicentric cohorts are planned to confirm the results obtained here.

In conclusion, despite possible limitations (e.g., retrospective design, lack of surgical and survival outcomes due to a 121-month follow-up time, lack of independent review), this study provides very encouraging findings on the higher pCR rate and favorable safety profile for THP compared to ATH regimen in clinical practice for HER2-positive EBC, opening the way for additional analysis on larger cohorts of patients to further confirm the strength of these results.

## 5. Conclusions

Our observational study confirms a good activity profile in terms of pCR rate and a good safety profile in favor of THP compared to the ATH regimen as neoadjuvant treatment of HER2-positive EBC. Thus, our findings suggest that THP can be considered an effective strategy of treatment as an alternative to anthracycline-based regimens, which may be particularly useful in real-world scenarios.

## Figures and Tables

**Table 1:** Chemotherapy regimens: (**a**) sequential anthracyclines and taxanes plus trastuzumab (ATH); (**b**) pertuzumab, trastuzumab and docetaxel (THP).

(**a**)		
ddEC q2w x4	Docetaxel q2w x6	S
(E: 90 mg/mq; C: 600 mg/mq)	(60 mg/mq)	U
		R
		G
	Trastuzumab q2w x6	E
	(6mg/kg → 4 mg/kg)	R
		Y
(**b**)		
Docetaxel q3w x6		
(75 mg/mq)		S
		U
		R
Trastuzumab q3w x6		G
(8 mg/kg → 6 mg/kg)		E
		R
		Y
Pertuzumab q3w x6		
(840 mg → 420 mg)		

ddEC, dose-dense epirubicin plus cyclophosphamide; q2w, every 2 weeks; q3w, every 3 weeks.

**Table 2:** Patients’ characteristics in HER2-positive population according to the treatment received.

	Overall (n = 47) N (%)	THP (n = 23) N (%)	ATH (n = 24) N (%)	χ2 Test
Type of intravenous/subcutaneous adjuvant therapy				
None	3 (6.4)	0 (0.0)	3 (12.5)	*p* < 0.001
Pertuzumab/trastuzumab	16 (34.0)	16 (69.6)	0 (0.0)	
Trastuzumab	23 (48.9)	2 (8.7)	21 (87.5)	
TDM-1	5 (10.6)	5 (21.7)	0 (0.0)	
Age				
Median	49	49	49	*p* = 1.000
Range	34–79	34–79	38–66	
<40 yo	5 (10.6)	2 (8.7)	3 (12.5)	
≥40 yo	42 (89.4)	21 (91.3)	21 (87.5)	
Menopausal status				
Premenopausal	28 (59.6)	13 (56.5)	15 (62.5)	*p* = 0.770
Postmenopausal	19 (40.4)	10 (43.5)	9 (37.5)	
ECOG PS *				
0	44 (93.6)	22 (95.7)	22 (91.7)	*p* = 1.000
1	3 (6.4)	1 (4.3)	2 (8.3)	
CIRS **				
Primary	35 (74.5)	20 (87.0)	15 (62.5)	*p* = 0.138
Intermediate	11 (23.4)	3 (13.0)	8 (33.3)	
Secondary	1 (2.1)	0 (0.0)	1 (4.2)	
Cardiovascular comorbidities				
Yes	10 (21.3)	3 (13)	7 (29.2)	*p* = 0.177
No	37 (78.7)	20 (87)	17 (70.8)	
ER/PgR status				
Negative	21 (44.7)	9 (39.1)	12 (50.0)	*p* = 0.454
Positive	26 (55.3)	14 (60.9)	12 (50.0)	
Histological classification				
Ductal	42 (89.4)	19 (82.6)	23 (95.8)	*p* = 0.441
Lobular	3 (6.4)	2 (8.7)	1 (4.2)	
Other	2 (4.2)	2 (8.6)	0 (0.0)	
Grading				
2	21 (44.7)	14 (60.9)	7 (29.2)	*p* = 0.029
3	26 (55.3)	9 (39.1)	17 (70.8)	
Ki-67				
<20%	11 (23.4)	8 (34.8)	3 (12.5)	*p* = 0.093
20% or more	36 (76.6)	15 (65.2)	21 (87.5)	
T				
1	1 (2.1)	1 (4.3)	0 (0.0)	*p* = 0.242
2	26 (55.3)	13 (56.5)	13 (54.2)	
3	7 (14.9)	5 (21.7)	2 (8.3)	
4	13 (27.7)	4 (17.4)	9 (37.5)	
N				
0	9 (19.1)	4 (17.4)	5 (20.8)	*p* = 0.032
1	28 (59.6)	18 (78.3)	10 (41.7)	
2	9 (19.1)	1 (4.3)	8 (33.3)	
3	1 (2.1)	0 (0.0)	1 (4.2)	
Type of breast surgery				
Quadrantectomy	22 (46.8)	11 (47.8)	11 (45.8)	*p* = 1.000
Mastectomy	25 (53.2)	12 (52.2)	13 (54.2)	
Type of axillary surgery				
Sentinel node biopsy	35 (74.5)	23 (100)	12 (50)	*p* < 0.001
Axillary dissection	12 (25.5)	0	12 (50)	

* ECOG PS: Eastern Cooperative Oncology Group Performance Status. ** CIRS: Cumulative Illness Rating Scale.

**Table 3:** Predictive factors of pCR in HER2-positive patients.

	Non-pCR (n = 28) N (%)	pCR (n = 19) N (%)	
ER-PgR status			
Negative	9 (32.1)	12 (63.2)	*p* = 0.036
Positive	19 (67.9)	7 (36.8)	
Neoadjuvant treatment			
Pertuzumab–trastuzumab–taxane	10 (35.7)	13 (68.4)	*p* = 0.028
Anthracycline–taxane	18 (64.3)	6 (31.6)	
PIK3CA			
Wild-type	22 (88.0)	8 (61.5)	*p* = 0.094
Mutant	3 (12.0)	5 (38.5)	

**Table 4:** Clinical features of PIK3CA mutant patients and variants detected.

N. Pt.	ER/PgR	Ki67	Neoadjuvant Treatment	Pathological Response	N345x	C420R	E542x	E545x	Q546x	H1047x	G1049x
1	neg.	<20%	ATH	pCR	WT	WT	WT	WT	WT	MUT	WT
2	neg.	≥20%	ATH	non-pCR	WT	WT	WT	MUT	WT	WT	WT
3	pos.	<20%	THP	non-pCR	WT	WT	WT	MUT	WT	MUT	WT
4	pos.	≥20%	ATH	non-pCR	MUT	WT	WT	WT	WT	WT	WT
5	pos.	<20%	THP	pCR	WT	WT	WT	WT	MUT	WT	WT
6	neg.	≥20%	THP	pCR	WT	WT	WT	WT	WT	MUT	WT
7	neg.	<20%	THP	pCR	WT	WT	WT	WT	WT	MUT	WT
8	neg.	≥20%	THP	pCR	WT	WT	WT	WT	WT	MUT	WT

Variants as follows: c.1033A>C, c.1034A>T, c.1034A>C, c.1035C>A, indistinguishable (N345x, exon 4); c.1258T>C (C420R, exon 7); c.1624G>A, c.1625A>G, c.1625A>T, indistinguishable (E542x, exon 9); c.1633G>A, c.1633G>C, c.1634A>C, c.1634A>G, c.1635G>C, c.1635G>T, indistinguishable (E545x, exon 9); c.1636C>A, c.1636C>G, c.1637A>C, c.1637A>G, c.1637A>T, indistinguishable (Q546x, exon 9); c.3139C>T, c.3140A>G, c.3140A>T, indistinguishable (H1047x, exon 20); c.3145G>C, c.3146G>C, indistinguishable (G1049x, exon 20).

**Table 5:** Safety data in HER2-positive patients, based on the treatment received.

	ATH (n = 24) N (%)				THP (n = 23) N (%)			
	G1	G2	G3	G4	G1	G2	G3	G4
Anemia	11 (45.8)	1 (4.2)	0 (0.0)	0 (0.0)	0 (0.0)	4 (17.4)	0 (0.0)	0 (0.0)
Neutropenia	1 (4.2)	3 (12.5)	4 (16.7)	2 (8.3)	0 (0.0)	0 (0.0)	0 (0.0)	0 (0.0)
Thrombocytopenia	1 (4.2)	0 (0.0)	0 (0.0)	0 (0.0)	0 (0.0)	0 (0.0)	0 (0.0)	0 (0.0)
Nausea	13 (54.2)	4 (16.7)	0 (0.0)	0 (0.0)	8 (34.8)	3 (13.0)	0 (0.0)	0 (0.0)
Vomiting	4 (16.7)	0 (0.0)	0 (0.0)	0 (0.0)	0 (0.0)	0 (0.0)	0 (0.0)	0 (0.0)
Diarrhea	6 (25.0)	4 (16.7)	1 (4.2)	0 (0.0)	10 (43.5)	6 (26.1)	2 (8.7)	0 (0.0)
Constipation	10 (41.7)	2 (8.3)	0 (0.0)	0 (0.0)	5 (21.7)	0 (0.0)	0 (0.0)	0 (0.0)
Dysgeusia	2 (8.3)	0 (0.0)	0 (0.0)	0 (0.0)	4 (17.4)	0 (0.0)	0 (0.0)	0 (0.0)
Anorexia	1 (4.2)	0 (0.0)	0 (0.0)	0 (0.0)	1 (4.3)	0 (0.0)	0 (0.0)	0 (0.0)
Asthenia	12 (50.0)	8 (33.3)	2 (8.3)	0 (0.0)	7 (30.4)	5 (21.7)	0 (0.0)	0 (0.0)
Mucositis	13 (54.2)	3 (12.5)	0 (0.0)	0 (0.0)	8 (34.8)	1 (4.3)	0 (0.0)	0 (0.0)
Skin toxicity	7 (29.2)	1 (4.2)	0 (0.0)	0 (0.0)	1 (4.3)	1 (4.3)	0 (0.0)	0 (0.0)
Neurotoxicity	4 (16.7)	0 (0.0)	0 (0.0)	0 (0.0)	4 (17.4)	0 (0.0)	0 (0.0)	0 (0.0)
Hypertransaminasemia	2 (8.3)	2 (8.3)	2 (8.3)	0 (0.0)	0 (0.0)	0 (0.0)	0 (0.0)	0 (0.0)

**Table 6:** Electrocardiographic data, echocardiographic data and clinical cardiological safety in HER2-positive patients, based on the treatment received.

	THP (n = 23) N (%)				ATH (n = 24) N (%)			
	G1	G2	G3	G4	G1	G2	G3	G4
Conduction anomalies	0 (0.0)	0 (0.0)	0 (0.0)	0 (0.0)	5 (20.8)	0 (0.0)	0 (0.0)	0 (0.0)
Valvular disfunction	0 (0.0)	0 (0.0)	0 (0.0)	0 (0.0)	6 (25.0)	0 (0.0)	0 (0.0)	0 (0.0)
Diastolic disfunction (I)	0 (0.0)	0 (0.0)	0 (0.0)	0 (0.0)	2 (8.3)	0 (0.0)	0 (0.0)	0 (0.0)
Tachycardia	0 (0.0)	0 (0.0)	0 (0.0)	0 (0.0)	3 (12.5)	0 (0.0)	0 (0.0)	0 (0.0)
Palpitations	0 (0.0)	0 (0.0)	0 (0.0)	0 (0.0)	14 (58.3)	0 (0.0)	0 (0.0)	0 (0.0)
Dyspnea	0 (0.0)	0 (0.0)	0 (0.0)	0 (0.0)	8 (33.3)	0 (0.0)	0 (0.0)	0 (0.0)
Hypertension	0 (0.0)	0 (0.0)	0 (0.0)	0 (0.0)	3 (12.5)	0 (0.0)	0 (0.0)	0 (0.0)
Pericardial effusion or IVS * hypokinesia	0 (0.0)	0 (0.0)	0 (0.0)	0 (0.0)	2 (8.3)	0 (0.0)	0 (0.0)	0 (0.0)
Basal pBNP **								
Median	16				15.5			
Range	10–54				10–60			
Intermediate pBNP								
Median	10				15.5			
Range	10–41				10–70			
Final pBNP								
Median	10				13			
Range	10–39				10–46			
Basal EF *** (%)								
Median	60				65			
Range	55–73				56–76			
Intermediate EF (%)								
Median	60				64			
Range	55–71				52–78			
Final EF (%)								
Median	60				60			
Range	54–71				50–70			

IVS *: interventricular septum, pBNP **: pro-Brain Natriuretic Peptide, EF ***: Ejection Fraction.

## Data Availability

The data presented in this study are available upon request from the corresponding author.

## Abbreviations

HER2 = human epidermal growth factor receptor 2; EBC = early breast cancer; pCR = pathological complete response; ATH = anthracycline and taxanes plus trastuzumab; THP = pertuzumab, trastuzumab and docetaxel; FDA = Food and Drug Administration; PIK3CA = phosphatidylinositol-4,-5-bisphosphate 3-kinase catalytic subunit alpha; PI3Kα = phosphoinositide 3-kinase α; mTOR = mammalian target of rapamycin; RTKs = receptor tyrosine kinases; ER = estrogen receptors; DFS = disease-free survival; IHC = immunohistochemistry; FISH = fluorescence in situ hybridization; AEs = adverse events; NCI-CTC = National Cancer Institute Common Terminology Criteria; FFPE = formalin-fixed, paraffin-embedded; LOD = limit of detection; ECOG PS = Eastern Cooperative Oncology Group Performance Status; CIRS = Cumulative Illness Rating Scale; N = lymph nodes; T = the size of the main tumor; TDM-1 = trastuzumab emtansine; PgR = progesterone receptor; mut = mutant; wt = wild-type; G = grade; IVS = interventricular septum; pBNP = pro-Brain Natriuretic Peptide; EF = Ejection Fraction; LVEF = left ventricular ejection fraction; LVSD = left ventricular systolic dysfunction.
